# Severity of COVID-19 Patients Predicted by Serum Sphingolipids Signature

**DOI:** 10.3390/ijms221910198

**Published:** 2021-09-22

**Authors:** Enrica Torretta, Micaela Garziano, Mariacristina Poliseno, Daniele Capitanio, Mara Biasin, Teresa Antonia Santantonio, Mario Clerici, Sergio Lo Caputo, Daria Trabattoni, Cecilia Gelfi

**Affiliations:** 1 IRCCS Istituto Ortopedico Galeazzi, 20161 Milan, Italy; enrica.torretta@grupposandonato.it; 2 Dipartimento di Fisiopatologia Medico-Chirurgica e dei Trapianti, Università degli Studi di Milano, 20122 Milan, Italy; micaela.garziano@unimi.it (M.G.); mario.clerici@unimi.it (M.C.); 3 Dipartimento di Scienze Biomediche e Cliniche “L. Sacco”, Università degli Studi di Milano, 20157 Milan, Italy; mara.biasin@unimi.it (M.B.); daria.trabattoni@unimi.it (D.T.); 4 Unit of Infectious Diseases, Department of Clinical and Experimental Medicine, University of Foggia, 71122 Foggia, Italy; polisenomc@gmail.com (M.P.); teresa.santantonio@unifg.it (T.A.S.); sergio.locaputo@unifg.it (S.L.C.); 5 Department of Biomedical Sciences for Health, University of Milan, 20090 Segrate, Italy; daniele.capitanio@unimi.it; 6 Don C. Gnocchi Foundation, IRCCS, 20148 Milan, Italy

**Keywords:** COVID-19, COVID-19 severity, sphingolipids, acid sphingomyelinase, serine palmitoyltransferase, caspase 3, frailty, mass spectrometry

## Abstract

The reason behind the high inter-individual variability in response to SARS-CoV-2 infection and patient’s outcome is poorly understood. The present study targets the sphingolipid profile of twenty-four healthy controls and fifty-nine COVID-19 patients with different disease severity. Sera were analyzed by untargeted and targeted mass spectrometry and ELISA. Results indicated a progressive increase in dihydrosphingosine, dihydroceramides, ceramides, sphingosine, and a decrease in sphingosine-1-phosphate. These changes are associated with a serine palmitoyltransferase long chain base subunit 1 (SPTLC1) increase in relation to COVID-19 severity. Severe patients showed a decrease in sphingomyelins and a high level of acid sphingomyelinase (aSMase) that influences monosialodihexosyl ganglioside (GM3) C16:0 levels. Critical patients are characterized by high levels of dihydrosphingosine and dihydroceramide but not of glycosphingolipids. In severe and critical patients, unbalanced lipid metabolism induces lipid raft remodeling, leads to cell apoptosis and immunoescape, suggesting active sphingolipid participation in viral infection. Furthermore, results indicated that the sphingolipid and glycosphingolipid metabolic rewiring promoted by aSMase and GM3 is age-dependent but also characteristic of severe and critical patients influencing prognosis and increasing viral load. AUCs calculated from ROC curves indicated ceramides C16:0, C18:0, C24:1, sphingosine and SPTLC1 as putative biomarkers of disease evolution.

## 1. Introduction

The 2019 coronavirus disease (COVID-19) is an ongoing pandemic that started in December 2019 in Wuhan (China), caused by severe acute respiratory syndrome coronavirus 2 (SARS-CoV-2). To date (August 2021), more than 4.4 million people worldwide have died from COVID-19 infection, and mortality risk increases in subjects with cardiovascular diseases, high blood pressure, diabetes, and metabolic syndrome. Furthermore, a high percentage of patients suffer from persistent symptoms and comorbidities associated with organ damage with long-term effects that remain unknown. The reason behind such a high inter-individual variability in response to SARS-CoV-2 infection is poorly understood, as well as the possible patient outcome. The majority of patients experience mild or moderate symptoms with a good prognosis [1]. However, about 20% of patients progress into severe or critical phase, suffering from severe respiratory failure, which requires mechanical ventilation and intensive care unit admission [2,3]. Disease severity is determined based on a set of clinical characteristics such as respiratory rate (≥30 breaths/min), mean oxygen saturation (≤94% in the resting state), or arterial blood oxygen partial pressure/oxygen concentration (≤300 mmHg). It is clear that prognostic markers of severity and possible predictors of patient outcomes are needed.

The virus infection occurs through the binding of viral Spike proteins (S-protein) to human cells utilizing a 2-step process that involves Angiotensin-Converting Enzyme-2 (ACE2) and Transmembrane Serine Protease (TMPRSS)-2. Once bound to ACE, the virus triggers the processing of ACE-2 through the ADAM-17/TNF-alpha-converting enzyme that induces the cleavage of ACE2’s extracellular domain and internalization. Next, through the assistance of clathrin [4], viral particles and host cells fuse, and the intracellular structure of ACE2 aids viral transport from the cell membrane to the cytoplasm, developing SARS. Another way of SARS-CoV-2 entry is by a membrane serine protease TMPRSS2 that proteolytically cleaves and activates viral envelop glycoproteins. Recently a selective advantage in lung cells and primary airway epithelial cells has been attributed to TMPRSS2 that enables endosome-independent virus entry through the furin polybasic cleavage site without endosomal acidification, making chloroquine treatment ineffective [4,5,6,7,8].

Giving ACE2 and TMPRSS2 as specific targets of COVID-19, the plasma membrane and the lipid raft in which ACE2 and TMPRSS2 are embedded can actively participate in viral infection [9,10]. The lipid raft is enriched in sphingolipids. The latter is a bioactive class of amphipathic molecules involved in a number of biological functions such as inflammation, immune cell activation, and recognition of exogenous agents. Defects in sphingolipid metabolism are involved in the pathogenesis of pulmonary infections and several respiratory diseases (asthma, cystic fibrosis, chronic obstructive pulmonary disease) [11,12,13]. Recent papers described that viruses induce significant remodeling of lipid composition in host cellular membranes [14,15,16,17,18], and sphingolipid changes associated with viral infections have been reported in plasma or serum [19,20,21]. Furthermore, studies in freshly isolated human nasal epithelial cells of COVID-19 positive volunteers demonstrated that acid sphingomyelinase (aSMase) and ceramide C16:0 promote viral spreading and treatment with aSMase inhibitors such as ambroxol or amitriptyline almost completely inhibit the viral infection [22,23].

The present study aims to unravel, in serum, sphingolipids associated with membrane disturbances that can lead to viral load increase. Furthermore, we would like to get a better insight into the panel of sphingolipids characterizing SARS-CoV-2 affected patients undergoing severe illness with the final goal of identifying putative biomarkers able to predict COVID-19 disease evolution but also contributing to define which species are involved in viral load increase. 

In this study, to overcome dynamic range limitations imposed by this class of molecules in serum [24], a combination of untargeted and targeted lipidomics was performed to investigate sphingolipid levels in a cohort of 83 subjects that included 24 healthy controls and 59 COVID-19 patients with different disease severity (i.e., mild, moderate, severe, and critical). The sphingolipid profile was correlated with clinical parameters. Results indicate a positive correlation for some sphingolipid species and no correlation for some others considered as protective. Furthermore, results indicated that the sphingolipid profile might be linked to disease severity, highlighting the relevance of monitoring this class of molecules to predict patient’s outcome and proposing specific sphingolipid molecules to be targeted for treating COVID-19 patients.

## 2. Results

### 2.1. Biochemical Parameters Assessment

Anthropometrics and the biochemical assessment are summarized in Table 1 and in Table S1. Patients (pts) were 33 men (56%) and 26 women (44%), mean age 57 (±17) years. Hypertension (24 pts, 40%), diabetes (9 pts, 15%), dyslipidemia (8 pts, 13.5%), solid/hematologic tumors (4 pts, 7%), obesity (4 pts, 7%), COPD (3 pts, 5%,) were the major comorbidities detected. To note, two patients presented syphilis and HBV coinfection, respectively. Blood parameters were collected at admission.

A significant elevation in inflammation markers (IL-6: 55 ± 97 pg/mL; PCR: 65 ± 74 mg/dL; D-dimers: 2258 ± 5062 ng/mL; Ferritin: 446 ± 410 ng/mL; LDH: 290 ± 197 UI/L), along with an abnormal blood cell count (HB 12 ± 2 gr/dL; WBC 6.1 ± 5.5 × 10^3^ cells/mm^3^; PLT 235 ± 112 × 10^3^ cells/mm^3^) were reported. Normal liver and kidney function were overall detected (GOT: 36 ± 40 mU/mL; GPT: 39 ± 64 mU/mL GGT: 65 ± 129 UI/L; serum creatinine:1.01 ± 6 mg/dL).

Hydroxychloroquine was prescribed to 47 pts (80%), 22 of whom were treated with a Protease Inhibitor (Lopinavir/RTV, 9 pts, or Darunavir/COBI, 10 pts), in line with the standard of care at the time of the patient’s hospitalization. Ten pts (17%) also received Tocilizumab in combination with one of the aforementioned treatments. Seven pts (12%) did not receive any therapy. 

Oxygen was administered to 44 pts (75%), the majority of whom (27 pts, 61%) received oxygen therapy in a Venturi Mask. Six pts (14%) required High Flow Nasal (HFN) oxygen support, and 5 (11%) noninvasive ventilation (NIV). Seven pts (12%) with severe SARS-CoV-2 pneumonia were transferred to the ICU; 3 of them received mechanical ventilation.

Death was reported in 4 pts (7%), all with moderate to critical disease severity, while the remaining patients were discharged after clinical and/or virological recovery.

### 2.2. Serum Sphingolipid Levels Changes in COVID-19

**Figure 1:**
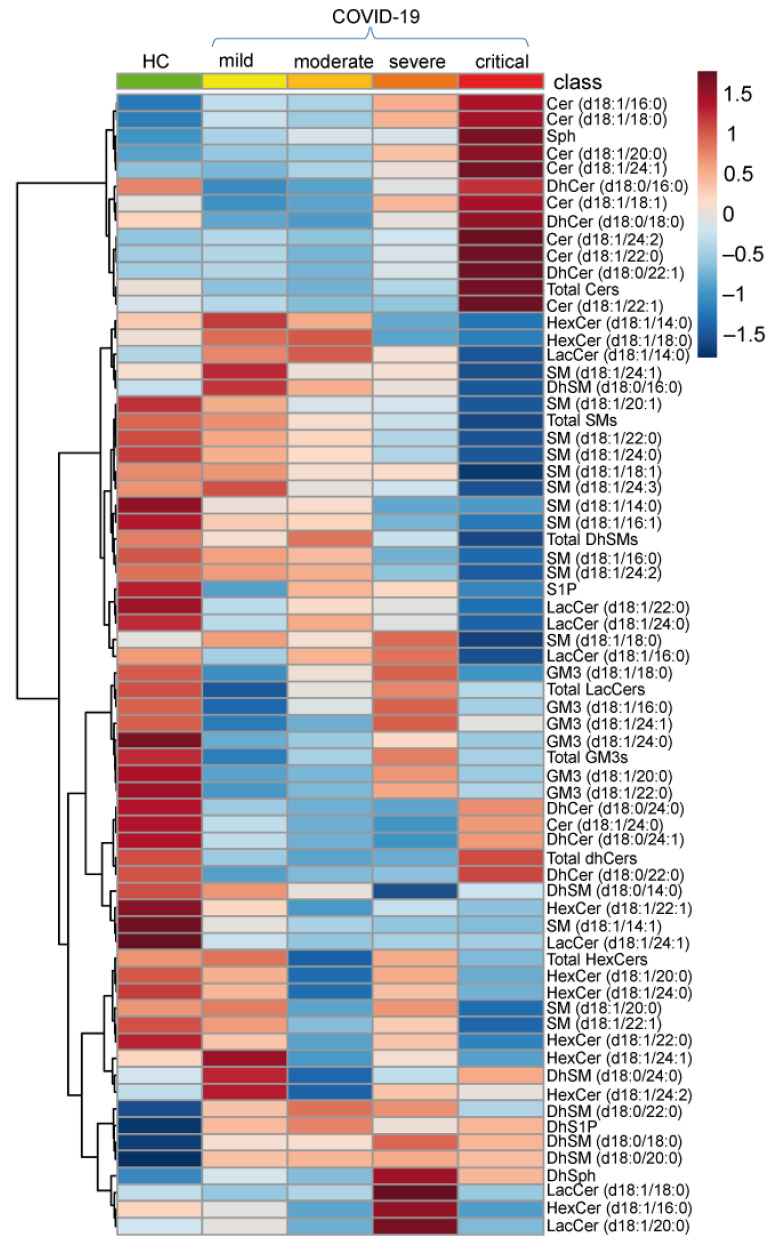
Heatmap of hierarchical clustering of the 68 sphingolipids quantified by LC-MS/MS in healthy controls (HC) and in COVID-19 patients (mild, moderate, severe, critical). Sphingolipids’ average abundances for each class are displayed.

Sphingolipid serum levels in COVID-19 patients were investigated by untargeted and targeted lipidomics. About 60 species including ceramides (Cers), dihydroceramides (DhCers), sphingomyelins (SMs), dihydrosphingomyelins (DhSMs), hexosylceramides (HexCers), lactosylceramides (LacCers), monosialodihexosyl gangliosides (GM3s), sphingosine (Sph), dihydrosphingosine (DhSph), sphingosine-1-phosphate (S1P) and dihydrosphingosine-1-phosphate (DhS1P) were quantified in mild, moderate, severe and critical COVID-19 patients, as well as in healthy subjects (HC). Figure 1 shows the average values of each sphingolipid species in all groups, revealing a considerable remodeling of serum sphingolipid species in COVID-19 disease.

Differences in serum sphingolipid levels were assessed for each sphingolipid acyl-chain towards HC and among COVID severity groups. To facilitate reading, results are described following the synthetic/degradation sphingolipid route (Figure S1).

#### 2.2.1. De Novo Pathway Ceramide Precursor, Dihydrosphingosine (DhSph), Increased with Disease Severity Whereas Dihydroceramides (DhCer) Levels Were at Variance According with COVID-19 Patient Severity and Acyl-Chain Length

**Figure 2:**
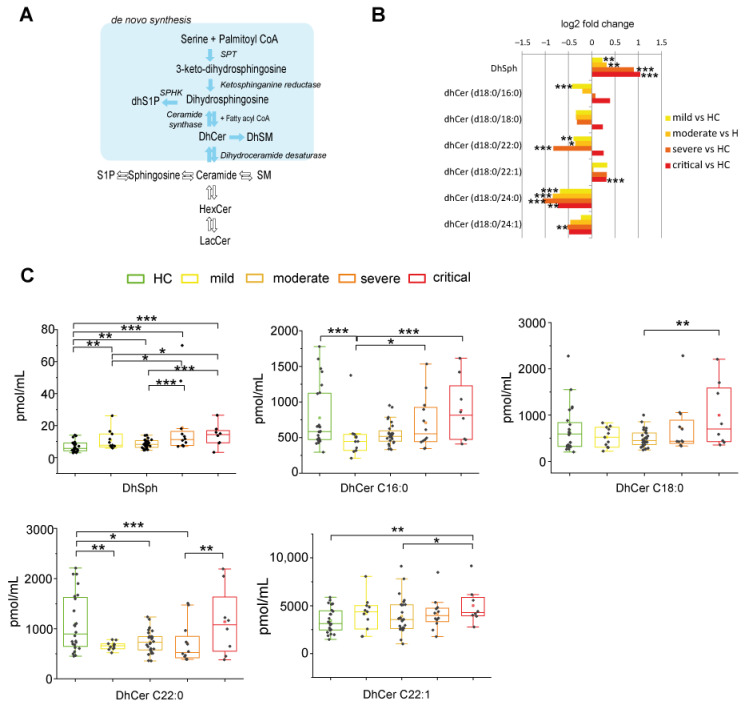
Serum sphingolipid changes related to COVID-19 in de-novo synthesis pathway. (**A**) Close up of de novo synthesis pathway, highlighted in light blue. (**B**) Log 2 fold changes in the median values of DhSph and DhCers in COVID-19 severity groups vs. healthy controls (HC). (**C**) Box plots of DhSph, DhCer C16:0, DhCer C18:0, DhCer C22:0, and DhCer C22:1 serum levels, according to COVID-19 severity. Box plots and whiskers represent the interquartile range with median value (central line) and the lowest and largest data point measured, respectively. Each measurement was performed in triplicate. Data were analyzed using Kruskal-Wallis ANOVA, followed by Dunn’s post hoc test for multiple comparisons. * *p*-value < 0.05, ** *p*-value < 0.01, *** *p*-value < 0.001.

DhSph and DhCer are the direct precursors of ceramide in the de-novo pathway (Figure 2A). Log2 fold changes were calculated to assess their levels in different groups compared to HC (Figure 2B). Mild and moderate patients showed higher levels of DhSph that further increased in severe and critical groups. Mild patients are characterized by lower levels of DhCer C16:0 compared to HC. DhCer C18:0 and C22:0 levels followed the same trend, were lower in mild, moderate, and severe patients compared to HC, but not in critical patients. All patients showed higher levels of C22:1 and lower levels of C24:0 and C24:1 compared to HC. Box plots in Figure 2C display differences according to disease severity. DhSph levels increased in severe and critical patients vs. mild and moderate. DhCer C16:0 levels increased progressively in severe and critical groups vs. mild patients. In the critical group, DhCer C18:0 and C22:1 levels increased compared to moderate, whereas DhCer C22:0 increased compared to severe patients, indicating that critical patients are characterized by DhCers increments irrespectively from their chain length. 

#### 2.2.2. Long Chain and Very Long Chain Ceramides Progressively Increased with Disease Severity except for Ceramide C24:0

**Figure 3:**
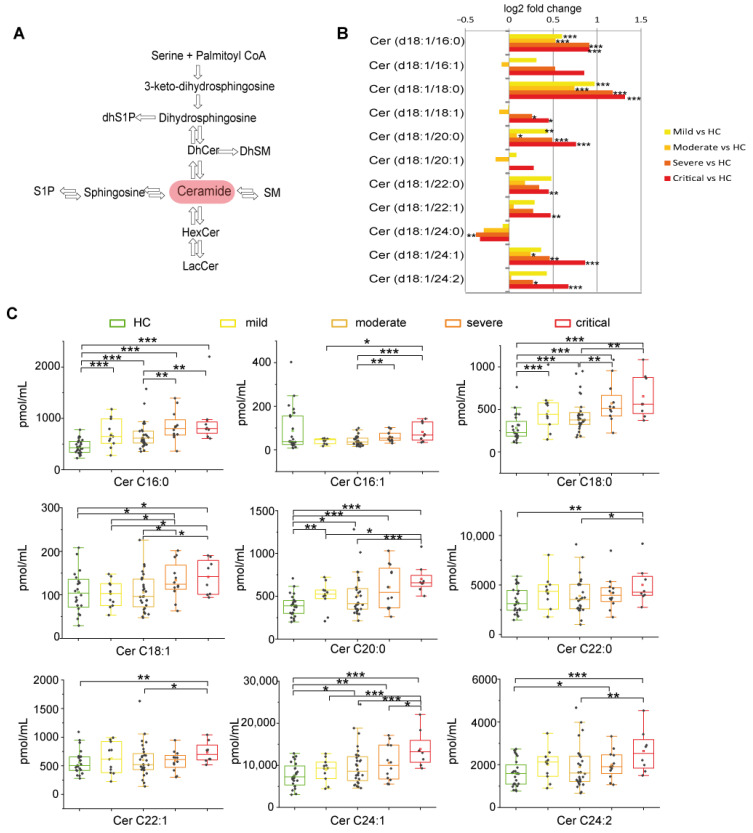
Ceramides changes in COVID-19. (**A**) Close up showing the central role of ceramide in the sphingolipid biosynthetic/catabolic pathway. (**B**) Log 2 fold changes in the median values of ceramides (C16:0, C16:1, C18:0, C18:1, C20:0, C20:1, C22:0, C22:1, C24:0, C24:1, C24:2) in COVID-19 severity groups vs. healthy controls (HC). (**C**) Box plots of serum ceramide species changed according to COVID-19 severity (C16:0, C16:1, C18:0, C18:1, C20:0, C22:0, C22:1, C24:1, C24:2). Each measurement was performed in triplicate. Data were analyzed using Kruskal-Wallis ANOVA, followed by Dunn’s post hoc test for multiple comparisons. * *p*-value < 0.05, ** *p*-value < 0.01, *** *p*-value < 0.001.

Ceramide is the hub molecule in sphingolipid metabolism (Figure 3A). Log2 fold changes showed higher levels of Cer C16:0, C18:0, and C20:0 in all disease stages. Cer C24:1 increased in moderate, severe, and critical, C18:1 and C24:2 increased in severe and critical patients, whereas C22:0 and C22:1 increased in critical patients only. Cer C24:0 is the solely decreased species (Figure 3B). Severe and critical groups differed from moderate, having higher levels of C16:0, C16:1, C18:0 C18:1, and C24:1. Cer C20:0, C22:0, C22:1, and C24:2 increased in critical vs. moderate only. Mild patients were at variance, having lower levels of C16:1, C18:1, C20:0, and C24:1 compared to critical (Figure 3C). Collectively these results indicate that critical and severe patients are characterized by increments of Cers irrespectively from their chain length, except for Cer 24.0, which decreased.

#### 2.2.3. In the Catabolic Pathway, Sphingosine Increased with Disease Severity Whereas S1P Decreased in Severe and Critical Patients

**Figure 4:**
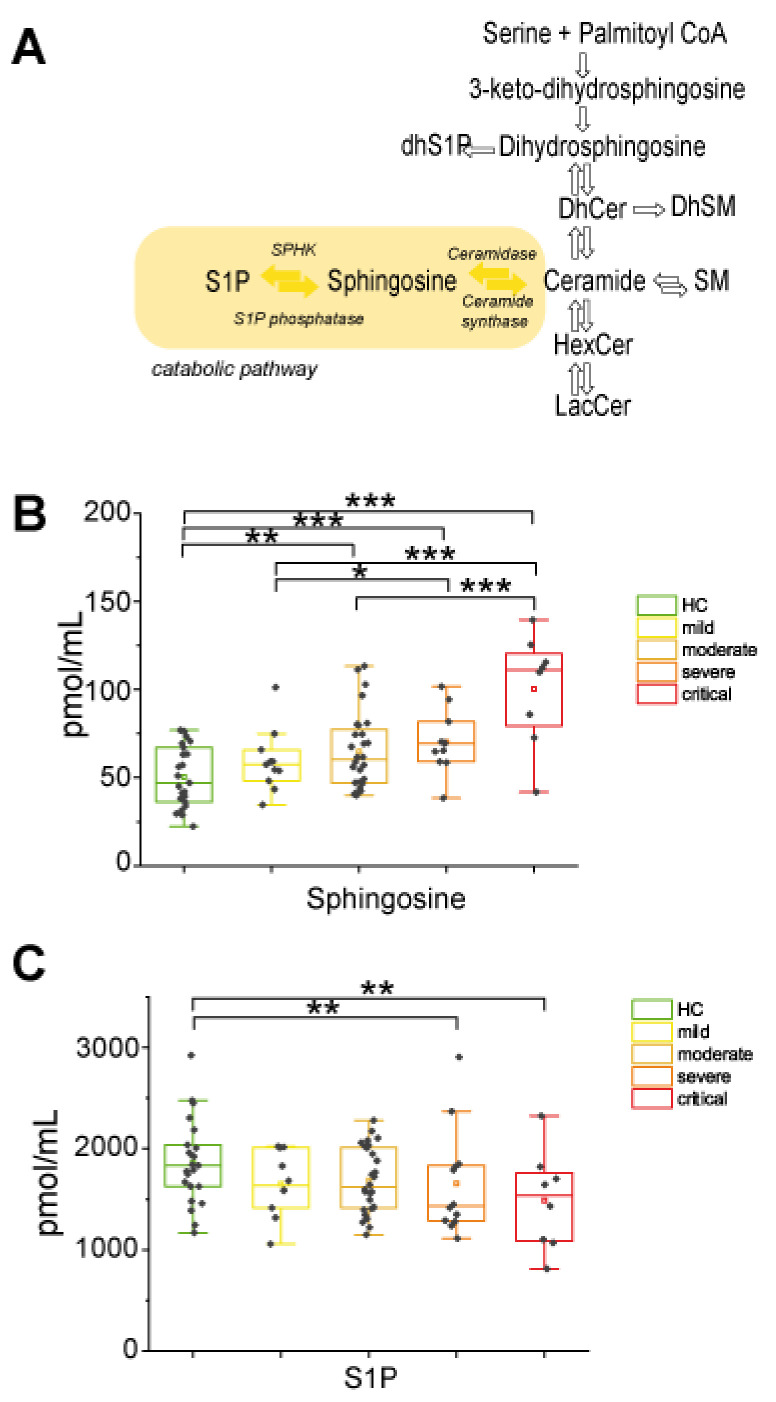
Sphingosine and sphingosine-1-phosphate changes in COVID-19. (**A**) Close up of the catabolic pathway, highlighted in yellow. (**B**) Box plots of sphingosine serum levels. (**C**) Box-plot of S1P serum levels. Each measurement was performed in triplicate. Data were analyzed using Kruskal-Wallis ANOVA, followed by Dunn’s post hoc test for multiple comparisons. * *p*-value < 0.05, ** *p*-value < 0.01, *** *p*-value < 0.001.

In the catabolic pathway (Figure 4A), sphingosine (Sph) showed a progressive statistically significant increment according to disease severity. Furthermore, levels were higher in severe and critical groups vs. mild and in critical compared to moderate groups (Figure 4B). In contrast, S1P levels decreased in severe and critical COVID-19 patients compared to controls (Figure 4C), suggesting that in severe and critical patients, Sph is not further metabolized to S1P but contributes to the Cers pool level increase. 

#### 2.2.4. Sphingomyelin Decreased Reaching Lowest Levels in Severe and Critical Patients

**Figure 5:**
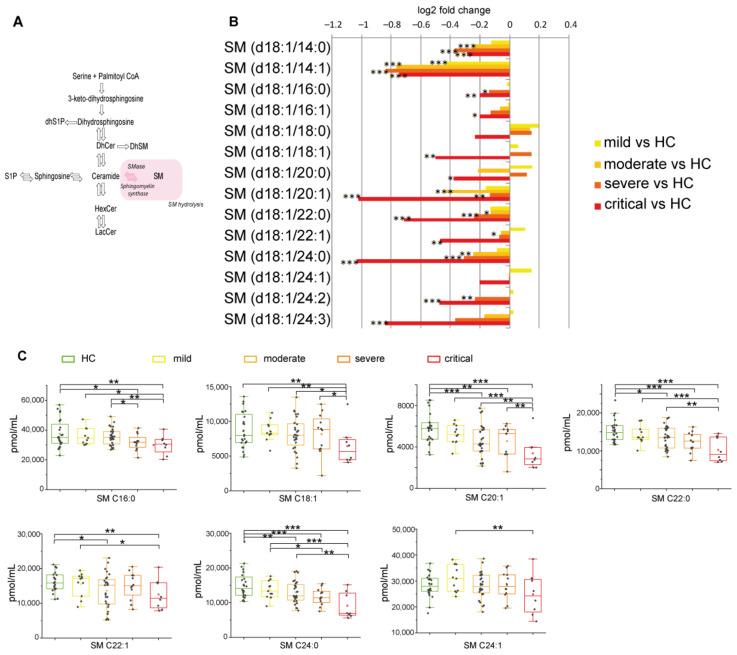
(**A**) Serum sphingolipid changes related to COVID-19 in the sphingomyelinase pathway, highlighted in pink. (**B**) Log 2 fold changes of the median values of SMs (C14:0, C14:1, C16:0, C16:1, C18:0, C18:1, C20:0, C20:1, C22:0, C22:1, C24:0, C24:1, C24:2, C24:3) in COVID-19 severity groups vs. healthy controls (HC). (**C**) Box plots of serum SM species changed according to COVID-19 severity (C16:0, C18:1, C20:1, C22:0, C22:1 C24:0, C24:1). Each measurement was performed in triplicate. Data were analyzed using Kruskal-Wallis ANOVA, followed by Dunn’s post hoc test for multiple comparisons. * *p*-value < 0.05, ** *p*-value < 0.01, *** *p*-value < 0.001.

In the sphingomyelinase pathway (Figure 5A), SMs decreased in COVID-19 patients compared to HC, with the lowest levels in severe and critical groups (Figure 5B). Critical patients display lower levels of C18:1 and C20:1 compared to severe. Furthermore, C16:0, C18:1, C20:1, C22:0, C24:0 were also lower compared to moderate and C16:0, C18:1, C20:1, C22:0, C22:1, C24:0, C24:1 were lower compared to mild (Figure 5C). Collectively these results indicate that SMs decreased, particularly in critical patients, irrespectively from their chain length.

#### 2.2.5. Glycosphingolipids and Disease Severity in COVID-19

**Figure 6:**
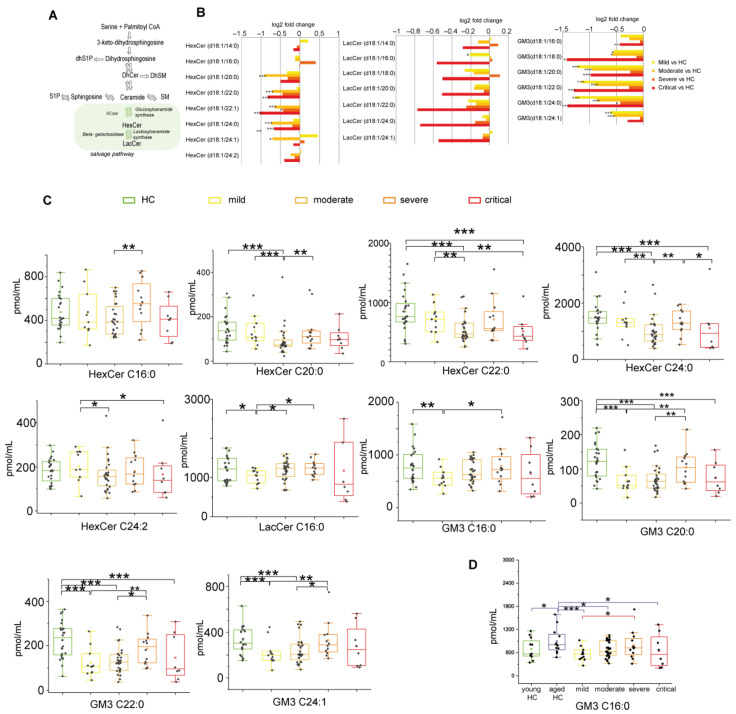
(**A**) Serum sphingolipid changes related to COVID-19 in the glycosphingolipid pathway, highlighted in green. (**B**) Log 2 fold changes of median values of HexCers (C14:0, C16:0, C20:0, C20:1, C22:0, C22:1, C24:0, C24:1, C24:2), LacCers (C14:0, C16:0, C18:0, C20:0, C22:0, C24:0, C24:1) and GM3s (C16:0, C18:0, C20:0, C22:0, C24:0, C24:1) in COVID-19 severity groups vs. healthy controls (HC). (**C**) Box plots of glycosphingolipid species changed according to COVID-19 severity (HexCers C16:0, C20:0, C22:0, C24:0, C24:2, LacCer C16:0, GM3s C16:0, C20:0, C22:0, C24:1). (**D**) Serum levels of GM3 C16:0 considering young HC and aged HC. Each measurement was performed in triplicate. Data were analyzed using Kruskal-Wallis ANOVA, followed by Dunn’s post hoc test for multiple comparisons. * *p*-value < 0.05, ** *p*-value < 0.01, *** *p*-value < 0.001.

Glycosphingolipids as hexosylceramides, lactosylceramides, and GM3s are involved in complex sphingolipid pathways (Figure 6A). In respect to HC, a statistical analysis indicated the lowest levels of HexCers and GM3s in critical and in moderate patients (for HexCers and GM3s). In mild, the decrement was observed for GM3s (Figure 6B). Mild patients showed higher levels of HexCer C20:0, C22, C24:0, C24:2, and GM3 C16:0, C20:0, C22:1, and C24:1 than the moderate group and lower levels of LacCer C16:0. Moderate patients displayed lower levels of HexCer C16:0, C20:0, C24:0, and GM3 C20, C22, and C24:1 compared to the severe group. Severe patients showed higher levels of HexCer 24:0, LacCer C18:0 compared to the critical group (Figure 6C). However, driven by a previous study [25] that indicated GM3s increase in relation to COVID-19 in young patients, data were re-analyzed considering HC as two classes, young HC, and aged HC vs. patients. GM3 C16:0, the most abundant GM3 in human serum, increased in aged HC, and the level was higher than in mild, moderate, and critical patients (data not shown) as indicated in panel D.

### 2.3. Acid Sphingomyelinase Levels Increased with COVID-19 Severity

**Figure 7:**
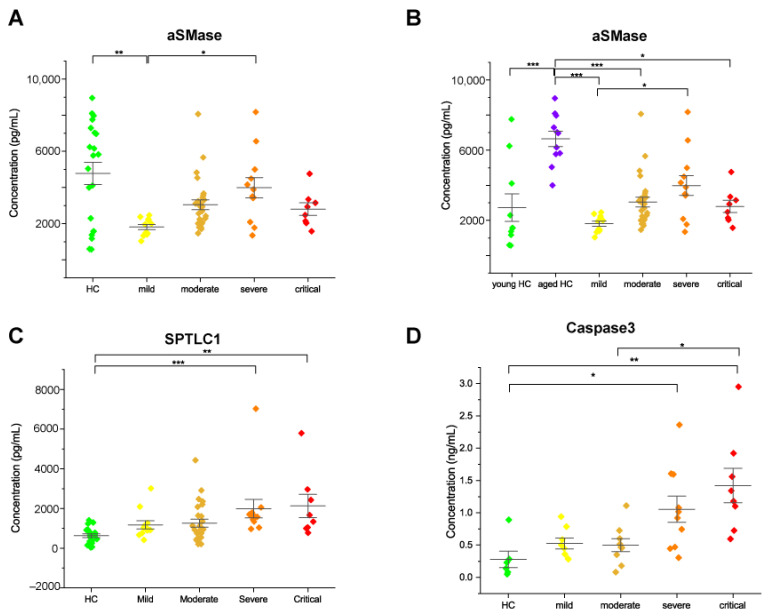
Scatter interval plots of acid SMase (**A**,**B**), serine palmitoyltransferase subunit 1 (SPTLC1) (**C**) and, caspase 3 (**D**) in serum of HC subjects, mild, moderate, severe, and critical patients, detected by ELISA assay. Each measurement was performed in duplicate. Data were analyzed using Kruskal-Wallis ANOVA, followed by Dunn’s post hoc test for multiple comparisons. * *p*-value < 0.05, ** *p*-value < 0.01, *** *p*-value < 0.001.

To further relate changes of Cer and SM specific chains in COVID-19 patients, levels of acid sphingomyelinase (aSMase) were assessed. This enzyme catalyzes the breakdown of SM to Cer and phosphorylcholine. Results from the ELISA assay showed a significant increase in acid SMase in severe compared to mild patients. (Figure 7A). This increment paralleled the increase in Cer and decrease in SM levels in relation to disease severity. An increasing trend was also observed in critical patients. By analyzing HC according to age, young HC vs. aged HC, we observed that levels of aSMase were increased in aged HC, and the enzyme level was comparable to severe patients (Figure 7B). Of note, three young subjects had aSMase levels comparable to aged subjects.

### 2.4. Serine Palmitoyltransferase and Caspase 3 Levels Increased in Severe and Critical Patients

To further support our results in relation to DhSph dysregulation and decreased levels of S1P in severe and critical patients, levels of serine palmitoyltransferase, which catalyzes the condensation of serine and palmitoyl CoA in the first step of the de-novo synthesis of sphingolipids were assessed. Serum levels of subunit 1 (SPTLC1) were quantified by the ELISA assay. Enzyme levels increased in severe and critical COVID-19 patients compared to healthy subjects (Figure 7C). To confirm cell death signaling in relation to an increase in pro-apoptotic species (ceramides and sphingosine) in COVID-19 patients, levels of Caspase 3 were assessed. Results showed an increase of Caspase 3 in severe and critical COVID-19 patients compared to healthy subjects and also in critical compared to moderate patients (Figure 7D).

### 2.5. Association of Serum Sphingolipids with Biochemical Parameters in COVID-19

**Figure 8:**
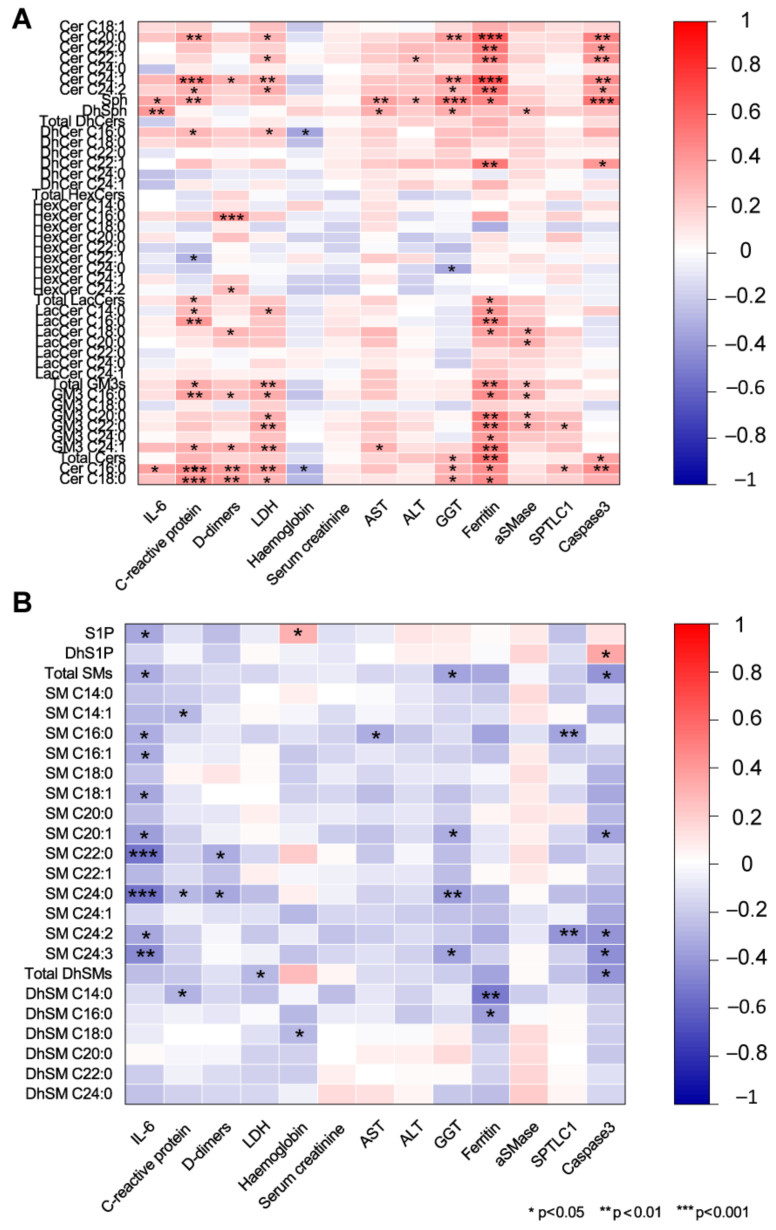
Spearman correlations between sphingolipid serum levels and circulating markers (IL-6, C-reactive protein, D-dimer, lactate dehydrogenase, hemoglobin, serum creatinine, aspartate aminotransferase, alanine aminotransferase, gamma-glutamyl transpeptidase, ferritin, and acid sphingomyelinase). (**A**) Ceramides, sphingosine, dihydrosphingosine, dihydroceramides, hexosylceramides, lactosylceramides, GM3s showed positive associations with all circulating markers except for hemoglobin, whereas (**B**) S1P, sphingomyelins, and dihydrosphingomyelins were negatively correlated. * *p*-value  <  0.05, ** *p*-value  <  0.01, *** *p*-value  <  0.001.

We are aware of the limitation of correlations since numbers are not the ones needed for a robust statistical significance; however, these results may represent a hint for further analyses in a larger cohort recruited from different hospitals. The possible association between sphingolipid levels and IL-6, CRP, ferritin, D-dimer, LDH, ALT, AST, and GGT was assessed by Spearman correlations (Figure 8). Cer C16:0 was positively associated with inflammatory markers IL-6, CRP, ferritin, with the coagulation functional marker D-dimer, with the marker of tissue damage LDH and with gamma-glutamyl transferase (GGT), whereas it was negatively correlated with hemoglobin. A similar behavior was observed for other acyl-chain Cers (C18:0, C20:0, C24:1, C24:2). At variance, Cer C24:0 did not show any correlation. Concerning DhCers, C16:0 negatively correlated with hemoglobin while C22:1 positively correlated with ferritin. Sph and DhSph positively correlated with IL6, CRP, AST, and GGT. Sph also correlated with ferritin. HexCer C16:0 showed a positive association with D-dimer, while HexCer C24:0 was negatively correlated with GGT. LacCers C14:0, C16:0, and C18:0 were positively associated with ferritin, and a positive association was also observed between LacCer C16:0 and CRP. GM3s C16:0 and C24:1 positive correlated with CRP, D-dimer, LDH, and ferritin. Total GM3s and GM3 C20:0, C22:0, C24:0 positively correlated with ferritin (Figure 8A). Conversely, S1P and SM behave differently. S1P, total SMs and SM (C16:0, C16:1, C18:1, C20:1, C22:0, C24:0, C24:2 and C24:3) were negatively correlated with IL-6. Furthermore, SM C22:0 and C24:0 were negatively correlated with D-dimer. SM C20:1, C24:0, C24:3 negatively correlated with GGT (Figure 8B). Acid SMase was positively correlated with LDH, creatinine, ALT, AST, ferritin, and with total GM3, GM3 C16:0, and C20:0. SPTLC1 was positively correlated with IL-6, LDH, AST and negatively with SM C16:0 and SM C24:2. Caspase 3 positively correlated with CRP, LDH, GGT, ferritin, DhCer C22:1, Sph, total Cers and Cers C16:0, C20:0, C22:0, C22:1, C24:1, and C24:2, whereas it was negatively correlated with total SMs, SM C20:1, C24:2, C24:3 and with total DhSMs. Considering the age of patients, Cer C16:0 and C24:1were positively correlated, whereas a significant negative correlation was observed for SM C22:0 and C24:0 and for DhSM C24:0 (Supplementary Table S2). 

### 2.6. Sphingolipids as Biomarkers for COVID-19 Disease

**Figure 9:**
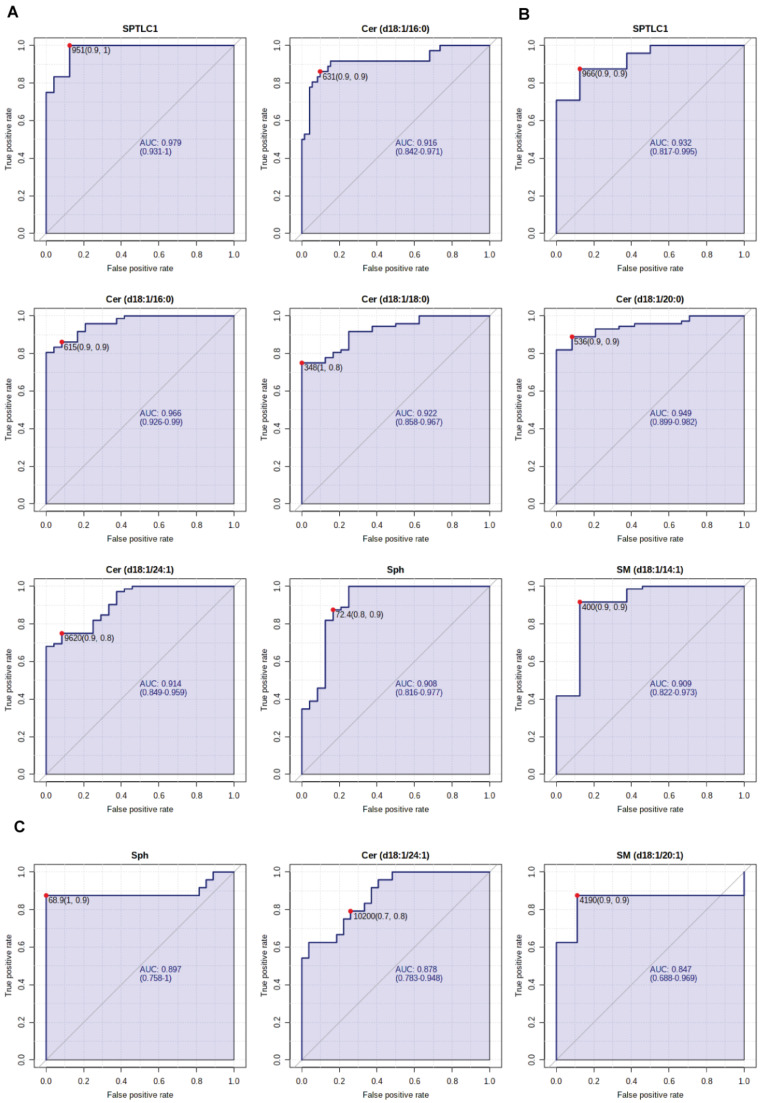
(**A**) ROC curve analyses of SPTLC1 and Cer C16:0 for patients with severe COVID-19 disease and HC subjects; (**B**) ROC curve analyses of SPTLC1, Cers C16:0, C18:0, C20:0, C24:1, Sph, SM C14:1 for patients with critical COVID-19 disease and HC subjects; (**C**) ROC curve analyses of Sph, Cer C24:1, SM C20:1 for patients with critical and mild COVID-19 disease. AUC values and 95% confidence intervals are reported, as well as cut-off values and sensitivity and specificity values in brackets.

Finally, receiver operating characteristic (ROC) curves were used to assess the diagnostic efficacy of each sphingolipid or enzyme here considered in discriminating severe and critical stages of COVID-19 disease from healthy controls and from milder stages of the disease. Comparing severe patients and HC, the area under the ROC curve (AUC) value for SPTLC1, was 0.97 (95% with a confidence interval (CI) of 0.931–1; *p* < 0.001) and for Cer C16:0 it was 0.916 (95% CI: 0.842–0.971; *p* < 0.0001) (Figure 9A). Comparing critical patients and HC, the AUC value for SPTLC1 was 0.927 (95% CI: 0.817–0.995; *p* < 0.001) and 0.966 (95% CI: 0.926–0.99; *p* < 0.0001) for Cer C16:0, respectively. For Cer C18:0 the AUC value was 0.922 (95% CI: 0.858–0.967, *p* < 0.0001), for Cer C20:0 was 0.949 (95% CI: 0.899–0.982; *p* < 0.0001) and 0.914, (95% CI: 0.849–0.959; *p* < 0.0001), for Cer C24:1, respectively. For Sph the AUC value were 0.908 (95% CI: 0.816–0.977; *p* < 0.0001) and 0.909 (95% CI: 0.822–0.973; *p* < 0.0001) for SM C14:1, respectively (Figure 9B). Comparing critical and mild patients, the AUC value for Sph was 0.897 (95% CI: 0.758–1; *p* < 0.0001), while for Cer C24:1 the AUC value was 0.886 (95% CI: 0.786–0.953; *p* < 0.0001) and 0.86 (95% CI: 0.715–0.975; *p* < 0.0001) for SM C20:1, respectively (Figure 9C). 

## 3. Discussion

In our study, sphingolipids were extracted from serum which contains extracellular vesicles, including exosomes and large plasma membrane-derived microvesicles released in the bloodstream; these vesicles transport proteins, lipids, and nucleic acids specific to the cell of origin. This untargeted/targeted sphingolipid analysis aimed to find changes in the sphingolipid composition, relate them with disease severity, and reveal new putative biomarkers to predict disease evolution toward severe conditions. Thus, the released or unreleased species from extracellular vesicles can provide a clue on the status of the plasma membrane and lipid raft according to disease severity and can provide hints or targets to counteract disease worsening and hypothesize new treatments. 

Results from the present study refer to 24 HC compared to 59 COVID-19 patients divided into four groups. Considering changes in respect to HC, the increment of DhSph, Sph, and of specific Cer chains and the progressive decrement of SMs, with lowest levels in critical patients, were observed. More specifically, results indicated that DhSph, Sph, Cers C16:0, C18:0, and C20:0 increased in all patients. Furthermore, Cer C18:1, Cer C24:1, increased in moderate and severe/critical patients, which, besides that, also showed a Cer C24:2 increase. Alongside the increase in Sph and Cers, a decrease in sphingosine-1-phosphate (S1P) was observed in severe and critical patients suggesting that the “sphingolipid rheostat” determining the cell fate is active [26]. In cells, ceramide and sphingosine are known to mediate apoptosis, cell cycle arrest, and differentiation, whereas S1P promotes proliferation and survival [27]. Our results collectively confirm a shift towards an apoptotic phenotype in severe and critical COVID-19 patients, as indicated by caspase 3 levels (Figure 3C), with a loss of the protective effect of S1P [28]. Van Echten-Deckert et al. described that the conversion of Sph to S1P can inhibit serine palmitoyltransferase activity [29]. Our results indicated that in severe and critical patients, increased levels of DhSph are related to the activation of serine palmitoyltransferase, confirmed by ELISA. The increase in palmitoyltransferase occurs in severe and critical patients and parallel the decrease in S1P, characteristic of these two groups of patients, leaving them without S1P protective signaling. The inhibition of this enzyme is a potential target to sustain S1P levels in line with the observation of Asao Katsume et al. [30]. Further studies at cellular levels are ongoing to better clarify our findings.

Results from this detailed lipidomic analysis suggest that disease severity is associated with a profound metabolic rewiring of the sphingolipid machinery that involves several species with different chain lengths. Of note, ELISA of aSMase, previously described in cellular studies [22,23], confirmed our results. Furthermore, together with aSMase, the study introduced for the first time the involvement of serine palmitoyltransferase associated with DhSph and S1P dysregulation. The increase in Caspase 3 confirmed the proapoptotic role of Cers species and Sph, promoting the negative evolution of the disease.

Results on Cer C24:0 indicated a decrease in moderate and severe/critical patients. It has been described that the ratio Cer C16:0/C24:0 (but also C18:0/C24:0 and C24:1/C24:0) is a predictor of cardiometabolic risk, whereas Cer C24:0 had negative or no correlation with adverse cardiac effects [31]. Thus, these results from previous studies can suggest a negative impact of the Cer C16:0/C24:0 ratio on the plasma membrane and lipid microdomains that can act as platforms for signal transduction affecting their organization and function [32].

The progressive increase in acid SMase affects Cers and GM3s levels, and the increase/decrease in specific chains appear to be associated with disease severity. aSMase is positively associated with Cer C16:0, Cer C18:0, total GM3, GM3 C16:0, GM3 C20:0, and LacCer (C18:0, C20:0) and negatively associated with Cer 24:0 suggesting that changed levels of these species can be correlated with disease evolution and possibly with an increased viral load [18]. Considering the age of patients, aSMase and GM3s were positively associated with severity and were age-dependent. These species were, in younger patients, not significantly changed in mild compared to aged-matched HC, while a progressive increase was observed in aged and severely affected patients. Furthermore, three young HC showed aSMase enzyme levels comparable to aged HC, suggesting that high levels of this enzyme can predict the subject’s frailty. Further quantitative studies have to be performed in a larger cohort to support our hypothesis. GM3 accumulation induces apoptosis through Caspase 3 activation, increases ROS production, and promotes cell senescence [33]. In melanoma cancer cells, GM3 increase is induced by aSMase, which is responsible for GM3 mediated apoptosis and an increase in gangliosides promotes immunoescape in these tumor cells [34]. In our patients, the increment of GM3 species directly correlates with increased levels of aSMase observed in severe patients. These results indicate that ganglioside levels and aSMase levels may trigger the negative evolution of disease prognosis, particularly in the elderly [35].

**Figure 10:**
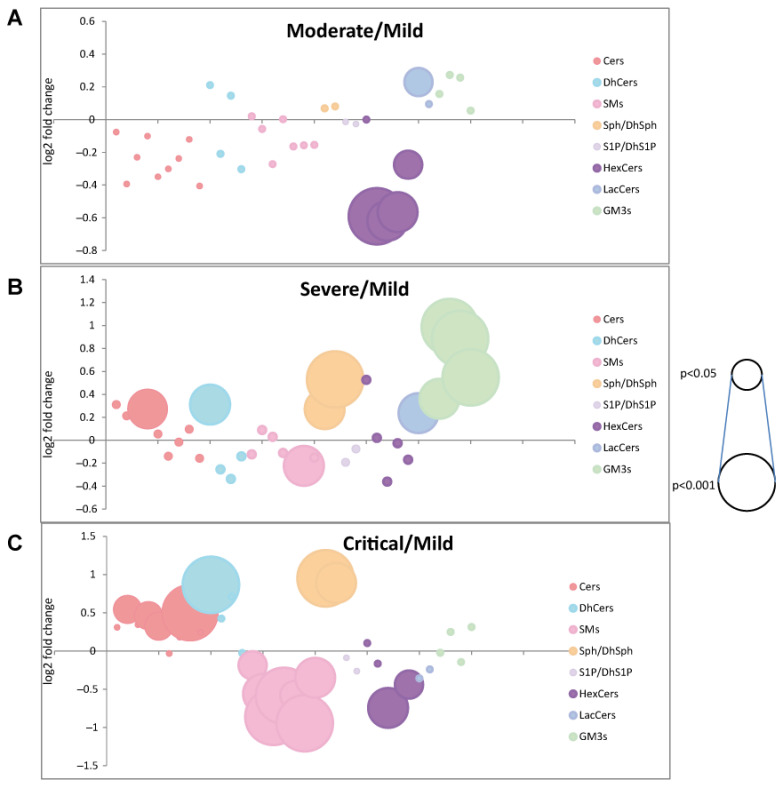
Bubble plots of log2 fold changes in abundance of sphingolipid species in serum from moderate (**A**), severe (**B**), and critical (**C**) patients compared to mild patients are shown. Bubble size represents the *p*-value from Kruskal Wallis ANOVA.

Results are summarized in Figure 10. 

Few studies assessed the sphingolipid plasma/serum profile in viral diseases. It has been investigated mainly in HCV and HIV infections. Results indicated that in the serum of acute and chronic HCV, a downregulation of Cer C24:0 and a simultaneous increase in the serum levels of Sph and DhSph were not revealed in HBV patients [15,19]. However, in HBV, the dysregulation of sphingolipids in serum can predict disease evolution toward acute-on chronic liver failure [21], indicating that levels of DhCer C24:0 can anticipate the negative patient’s outcome. In HIV patients, elevated plasma levels of C16:0 and C24:1 ceramides correlated with immune activation and inflammation and were associated with antiretroviral therapy and progression of carotid artery atherosclerosis [20]. In line with our results, changes in specific chains abundance appear to be a common trait of viral infections and of their outcome. The risk of death from COVID-19 is about 10 times higher in countries where most of the population is overweight (COVID-19 and Obesity: The 2021 Atlas) and previous studies from our group highlighted the role of acyl chain composition in bodily fluids in obesity and obesity-related comorbidities [36,37,38]. In obese subjects, high levels of total Cers, lower levels of total SM, and of DhSM compared to normolipidemic normal weight controls were found. Interestingly the same pattern also characterizes dyslipidemic normal weight subjects. Furthermore, a detailed study by A. M. Poss, based on machine learning and sphingolipid profile in sera, identified sphingosine, DhCer C16:0, DhCer C18:1, and Cer C18:0 as dysregulated sphingolipids chains that can contribute to predicting the evolution of CVD [39]. Based on our results, these profiles could also represent a predictive signature for a negative evolution of COVID disease, confirming the susceptibility of obese and CVD patients toward a bad prognosis in COVID infection. Another point to be considered is age, in which accumulation of specific chains of ceramide and decrease in SM have been described in plasma, and results on aged and AD subjects indicated that dysregulation of sphingolipid metabolism is associated with frailty [40,41]. Aged subjects with a background of dysregulated sphingolipid metabolism are more prone to develop a severe phenotype, as CVD patients and obese. In aged HC, our results, although in a restricted cohort, indicated higher levels of aSMase and GM3s that may represent a risk factor for developing a severe illness. Data from SLs serum profiling suggested that viral infections, aging, and obesity are characterized by lipid raft remodeling that impacts membrane structural properties and cell signaling. An increase in Cer and DhCer levels and decrease in Cer C24:0 can cause stiffness and change in cell membrane curvature, making cells more permeable to small and large size molecules [42]. In addition, Cers can promote transmembrane lipid motion [43]. Our patients are characterized by increased acid sphingomyelinase levels that, besides promoting an increase in GM3 mediated apoptosis, also promote the synthesis of long acyl chains ceramides at the expense of SM. This would generate unbalance on the structure of the membrane with an accumulation of cholesterol in the inner leaflet, as suggested by studies by Slotte and Bierman [44]. It has also been demonstrated that imbalance in sphingolipids composition in the surface area between two leaflets leads to spontaneous endocytosis [45], which could contribute to the negative outcome observed in severe and critical patients in which unbalance of ceramide long chains and SM occurs. Furthermore, progressive increase in LacCer, Cer(d18:1/18:1), DhCer (d18:0/16:0), Sph, DhSph, GM3 (d18:1/16:0), GM3 (d18:1/20:0), GM3 (d18:1/22:0), GM3 (d18:1/24:1) in severe patients compared to mild, may promote structural remodeling, apoptosis, and immunoescape. Collectively these results indicate an active participation of sphingolipids in viral infection. Treatment with aSMase inhibitors appears to decrease viral proliferation [46]. However, further studies of the sphingolipid profile after treatment with aSMase inhibitors would be required to precisely reveal which changes are induced in the serum sphingolipid profile and if treatments at the cell level can also promote a structural membrane lipid raft remodeling inhibiting virus entry in frail aged subjects.

Concerning the diagnostic potentiality of identified molecules, the present study suggests possible candidates as putative biomarkers of disease evolution based on ROC/AUC curves. Ceramides C16:0, C18:0, C24:1, sphingosine, and SPTLC1 can be considered as putative markers with ROC/AUC outstanding values comparing severe and critical patient vs. HC and excellent values in the comparison of mild to severe vs. critical patients. aSMase and GM3s do not, unfortunately, have such excellent or outstanding discriminatory power being age-dependent. A validation study on a larger independent cohort is needed, and it is currently ongoing.

Concerning correlations of sphingolipid composition and levels of clinical parameters, besides inflammatory markers (IL-6 and CRP), the positive association of ferritin with LacCer, GM3, Cer C16, Cer C18, Cer C20, DhCer, and Sph suggested an interplay among SLs acyl chains, iron metabolism and ROS production in COVID-19 evolution [47,48]. 

We believe that sphingolipid lipid composition has to be considered as a structural modifier that contributes to mechano-translate signals from lipid raft to cell membranes of endoplasmic reticulum, Golgi, lysosomes, and mitochondria, activating signaling processes. The identification of structural changes will represent a step forward not only in COVID 19 but also in other infectious or noninfectious disorders in which lipid transport is involved. 

In the past few months, other groups investigated the COVID-19 sphingolipid serum profile; however, the results were not always uniform. Our data on Sph and Cers levels are in agreement with studies based on virus inactivation by chloroform/methanol [19,21,25]. In other studies adopting viral heat inactivation results were at variance [49], emphasizing the importance of an extensive validation with standardized procedures for sample collection, sample pre-treatment, and lipid extraction to define a robust protocol that can cope with the interindividual variability avoiding confounder factors.

The major limitation of this study is the restricted number of subjects and the absence of a verification study through targeted lipidomics, which will require an independent group of subjects from a larger longitudinal cohort. Considering correlation results, in which a greater sample size is usually required, caution should be used. Finally, further mechanistic validations are needed and are currently ongoing to confirm conformational changes in plasma membranes.

## 4. Materials and Methods

### 4.1. Participants and Sample Collection

This study included a total of 59 COVID-19 positive patients (as determined by SARS-CoV-2 molecular test of nasopharyngeal swabs) hospitalized at Infectious Diseases Unit, Policlinic “Riuniti” of Foggia (Italy), from 1 March to 31 May 2020. According to COVID-19 disease severity, patients were divided into mild (11 pts, 19%), moderate (28 pts, 47%), severe (12 pts, 20%), and critical (8 pts, 13%), following the National Institute of Health (NIH) guidelines for COVID-19 treatment. Serum samples were collected at hospital admission and stored at −80 °C until use. Age and sex matched controls asymptomatic and COVID-19 molecular test negative subjects (N = 24) were included.

The present study conforms to the principles of the Helsinki Declaration and was approved by the Ethics Committee of Policlinic “Riuniti” of Foggia (protocol number 49/C.E./2021). All enrolled subjects provided their full informed consent. 

General characteristics and laboratory findings of enrolled subjects are summarized in Table 1 and in Table S1.

### 4.2. Reagents and Chemicals

LC-MS grade solvents and reagents as water, methanol, ammonium formate, formic acid, and acetate acid, as well as 3,5-Di-tert-4-butylhydroxytoluene (BHT), were from Sigma-Aldrich (Saint Louis, MO, USA). Ethanol and HPLC analytical grade chloroform were, respectively, from J.T. Baker (Center Valley, PA, USA) and Carlo Erba (Cornaredo, MI, Italy). Potassium hydroxide was from Merk Millipore (Burlington, MA, USA). Sphingosine (d17:1), sphinganine-1-phosphate (d17:1), ceramide (d18:1/12:0), sphingomyelin (d18:1/12:0) and glucosyl (β)ceramide (d18:1/12:0) were from Avanti Polar Lipids (Alabaster, AL, USA).

### 4.3. Lipid Extraction

Sera were managed in a Class II biosafety cabinet, and sphingolipids were extracted according to a previous study, with minor modification [38]. Briefly, 0.1 mL of serum was mixed with 0.1 mL of ultrapure water and 1.5 mL of methanol/chloroform 2:1, and fortified with internal standards 200 pmol: sphingosine (d17:1), sphingosine-1-phosphate (d17:1), ceramide (d18:1/12:0), sphingomyelin (d18:1/12:0), and glucosyl (β)ceramide (d18:1/12:0). Samples were briefly sonicated and heated at 48 °C overnight. Then, 0.15 mL of potassium hydroxide (KOH) 1 M in methanol was added to every sample, and after 2-h incubation at 37 °C, the solution was neutralized with 0.15 mL of acetic acid 1 M and dried with Speedvac. Samples were then resuspended in 0.5 mL of methanol and transferred to a clean Eppendorf tube. Samples were dried, resuspended in 0.15 mL of methanol, and centrifuged for 3 min at 10,000× *g*. Liquid phases were collected in UPLC glass vials and stored at −80 °C.

### 4.4. Untargeted Lipidomics

Liquid chromatography-mass spectrometer configuration included Waters Acquity UPLC system linked to a Q-TOF Synapt G2-Si (Waters, Milford, MA, USA). Ten µL of sphingolipid extract were injected and separated on a C8 Acquity UPLC BEH (Waters), 100 mm × 2.1 mm id, 1.7 µm, kept at 30 °C, using the following linear gradient: 0.0 min: 80% B; 3 min: 90% B; 6 min: 90% B; 15 min: 99% B; 18 min: 99% B; 20 min: 80% B, at 0.3 mL/min flow rate. Phase B consisted of 1 mM ammonium formate in methanol, 0.05 mM formic acid, while phase A was 2 mM ammonium formate in H_2_O, with 0.05 mM formic acid. The ESI ionization source was operated in positive ion mode with 30 V of sample cone voltage and 3.0 kV of capillary voltage. The desolvation temperature was set to 150 °C, and the ion source was 120 °C. The desolvation gas flow was set to 600 L/h. Data were captured under centroid mode, and the range of mass scan was set to 50–1500 Da. Accuracy and reproducibility were maintained employing an independent reference spray via LockSpray. Sphingolipids’ quantification was carried out using the ion chromatogram obtained for each compound using 50 mDa windows. The linear dynamic range was determined by the injection of standard mixtures. Positive identification of compounds was based on the accurate mass measurement, with an error <5 ppm and its retention time, compared to that of a standard (±2%). Mass spectra were analyzed by MassLynx™ 4.2 Software (Waters, Milford, MA, USA), and lipids were annotated as lipid subclasses as follows (sphingosine backbone/number of carbon atoms of the fatty acid: number of unsaturation of the fatty acid). MS/MS spectra were acquired, and the assignment of species was based on precursor ions and product ions m/z 264.268 and m/z 266.286, which correspond to sphingosine backbone (d18:1) and dihydrosphingosine backbone (d18:0), respectively.

### 4.5. Targeted Lipidomics

Sphingosine, dihydrosphingosine, S1P, and dihydroS1P were quantified using a Xevo TQ-S micro mass spectrometer (Waters, Milford, MA, USA). Sphingolipid extracts were injected and separated on a C8 Acquity UPLC BEH 100 mm × 2.1 mm id, 1.7 µm (Waters) kept at 30 °C, with the following gradient: 0.0 min: 80% B; 3 min: 90% B; 6 min: 90% B; 9 min: 99% B; 12 min: 99% B; 14 min: 80% B, at 0.3 mL/min flow rate. Phase A and phase B were the same as for untargeted lipidomics. An electrospray interface operating in positive ion mode was employed to obtain MS/MS spectra by acquiring MRM transitions of: sphingosine d17:1, 286.40 > 250.40, sphingosine d18:1, 300.40 > 264.40, sphingosine d18:0 302.4 > 266.4, cone voltage 40 V, collision energy 16 eV; sphingosine-1-phosphate d17:1, 366.40 > 250.40, sphingosine-1-phosphate d18:1, 380.40 > 264.40, sphingosine-1-phosphate d18:0 382.4 > 266.4, cone voltage 20 V, collision energy 16 eV. The capillary voltage was set at 3.5 kV. The source temperature was set to 150 °C. The desolvation gas flow was set to 1000, and the desolvation temperature was set to 350 °C. Data were acquired by MassLynx ™ 4.2 software and quantified by TargetLynx software.

### 4.6. Enzyme-Linked Immunosorbent Assays

Concentrations of acid sphingomyelinase, serine palmitoyltransferase (subunit 1), and caspase 3 in serum of patients were measured using Human Acid sphingomyelinase ELISA Kit (SMPD1) (Abcam, ab277075), SPTLC1 ELISA kit (AssayGenie, Dublin, Ireland), and Human Caspase 3 Instant ELISA kit (Thermo Fisher Scientific, Waltham, MA, USA), respectively, according to the manufacturer’s instructions. The absorbance was measured on a microplate reader (BioRad) using the wavelength of 450 nm. 

### 4.7. Statistical Analysis

Spearman correlations between clinical data and sphingolipid levels were performed with data from COVID-19 patients. Samples with missing clinical data were excluded from the calculations. Correlations between age and sphingolipid levels were performed for all the subjects. Correlation plots for all variables grouped by sphingolipid class were presented. Only correlations with r > 0.3 and *p*-value < 0.05 were indicated and further discussed. ROC curve analyses were performed by Metaboanalyst 5.0 to determine the diagnostic value of serum sphingolipids and enzymes in COVID-19 patients, considering the area under the ROC curve (AUC) with 95% confidence interval (CI), sensitivity, specificity, and cut-off value.

## Figures and Tables

**Table 1:** Clinical and biochemical assessments of study participants. Data were median (min-max) or n (%); *p*-values comparing the disease groups were calculated using Kruskal Wallis ANOVA test (age, hemoglobin, platelets, white blood cells, neutrophils, lymphocytes, interleukin-6, C-reactive protein, D-dimer, lactate dehydrogenase, serum creatinine, aspartate aminotransferase, alanine aminotransferase, gamma-glutamyl transpeptidase, ferritin) or the chi-squared test (sex, co-infections, comorbidities).

	Healthy Controls (*n* = 24)	Mild (*n* = 11)	Moderate (*n* = 28)	Severe (*n* = 12)	Critical (*n* = 8)	
Characteristics						*p*-value
Age, years	52 (26–78)	45 (19–85)	55 (24–98)	62.5 (40–84)	63.5 (49–75)	0.276
Sex						0.194
Men	11 (46%)	3 (27%)	16 (57%)	8 (67%)	6 (75%)	
Women	13 (54%)	8(73%)	12 (43%)	4 (33%)	2 (25%)	
Co-infections						
Hepatitis B	0	1 (9%)	0	0	0	
Syphilis	0	0	1 (3%)	0	0	
Co-morbidities						
Hypertension		5 (45%)	9 (32%)	6 (50%)	4 (50%)	0.643
Diabetes		2 (18%)	5 (18%)	2 (17%)	0	0.969
Dyslipidemia		2 (18%)	1 (4%)	5 (42%)	0	
Obesity		0	3 (11%)	1 (8%)	0	
Solid/hematologic tumors		0	1 (4%)	3 (25%)	0	
COPD		0	1 (4%)	1 (8%)	1 (12%)	
Death	0	1 (9%)	0	1 (8%)	2 (25%)	
Haemoglobin, g/dL		12.6 (10.3–17.2)	14.2 (8.7–17.5)	12.5 (10.2–14.2)	10.3 (8.1–14.4)	0.024
Platelets, ×10^3^ cells/mm^3^		198 (145–416)	209 (91–407)	256 (98–661)	251 (74–483)	0.66
White Blood Cells, ×10^3^ cells/mm^3^		3800 (1790–6650)	5390 (1460–9940)	6095 (1580–11,150)	6380 (3950–42,510)	0.0265
Neutrophils, ×10^3^ cells/mm^3^		3.745 (1.26–6.55)	3.14 (1.46–21.67)	4.265 (1.77–6.37)	4.97 (2.04–23.55)	0.261
Lymphocytes, ×10^3^ cells/mm^3^		1.15 (0.43–1.96)	1.165 (0.3–221)	0.79 (0.45–1.71)	1.07 (0.5–2.19)	0.281
Interleukin-6, pg/mL		3.21 (0.44–26.32)	10.615 (1.83–70.59)	120.72 (36.11–215.41)	126.27 (81.57–546.78)	<0.001
C-reactive protein, mg/dL		9.3 (0.3–86.1)	40.05 (0.8–320.1)	66.4 (7.1–254)	71.8 (1–176.7)	0.0387
D-dimer, ng/mL		488 (101–17,947)	772.5 (218–36,910)	1544 (421–5194)	557 (369–5309)	0.702
Lactate Dehydrogenase, UI/L		166 (114–275)	242 (54–941)	251 (137–1424)	361 (223–577)	0.0016
Serum creatinine, mg/dL		0.73 (0.58–1.43)	0.85 (0.57–1.31)	0.945 (0.42–4.39)	0.895 (0.52–2.12)	0.595
Aspartate Aminotransferase, mU/mL		21 (14–54)	22 (11–114)	20 (0.99–234)	43.5 (11–169)	0.07318
Alanine Aminotransferase, mU/mL		15 (9–66)	25 (7–158)	18 (8–103)	41 (6–439)	0.1933
Gamma-glutamyl transpeptidase, UI/L		25 (12–84)	27 (9–110)	21 (10–354)	97 (23–913)	0.00977
Ferritin, ng/mL		123 (11–378)	206 (15–1000)	647 (340–1000)	886 (140–1000)	0.01085

## Data Availability

The data presented in this study are available in Supplementary Tables. Study data from this human study other than those published in this work are under privacy regulations but can be obtained on a case-to-case basis upon reasonable request from the corresponding author.

## Supplementary Materials

The following are available online at https://www.mdpi.com/article/10.3390/ijms221910198/s1.
