# Supplementary material for: Severity of COVID-19 Patients Predicted by Serum Sphingolipids Signature
Source: Int J Mol Sci. 2021 Sep 22;22(19):10198. doi: 10.3390/ijms221910198 (PMC8508132; doi:10.3390/ijms221910198)
Supplement: Supplementary file 1 [file ijms-22-10198-s001.zip › Figure S1.pdf]

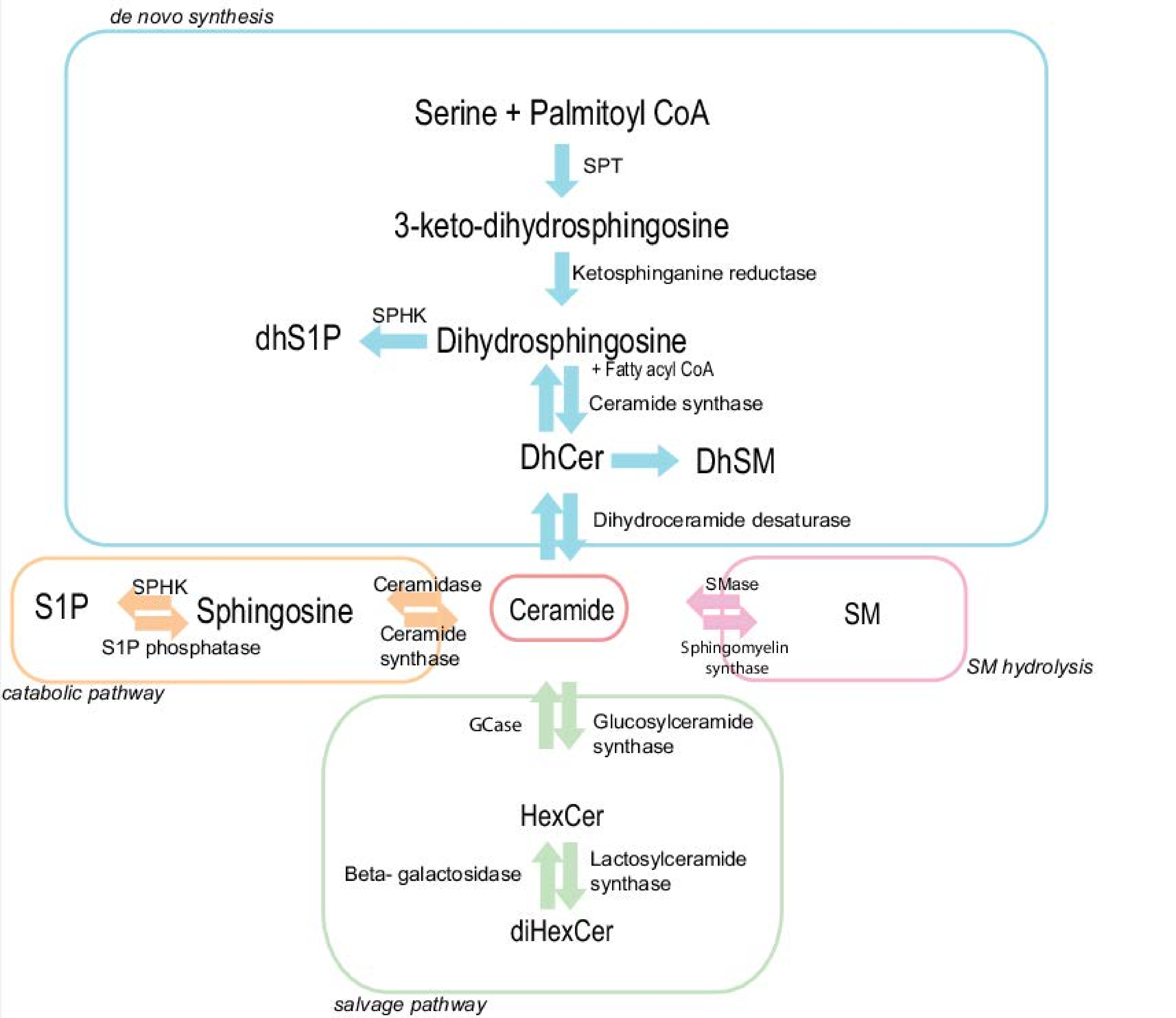
de novo synthesis
Serine + Palmitoyl CoA
SPT
3-keto-dihydrosphingosine
Ketosphinganine reductase
dhS1P
SPHK
Dihydrosphingosine
+ Fatty acyl CoA
Ceramide synthase
DhCer
DhSM
Dihydroceramide desaturase
S1P
SPHK
Sphingosine
S1P phosphatase
Ceramidase
Ceramide synthase
catabolic pathway
Ceramide
SMase
SM
Sphingomyelin synthase
SM hydrolysis
GCase
Glucosylceramide synthase
HexCer
Beta- galactosidase
Lactosylceramide synthase
diHexCer
salvage pathway
